# Diiodido(1,10-phenanthroline-κ^2^ *N*,*N*′)platinum(II)

**DOI:** 10.1107/S1600536809053100

**Published:** 2009-12-16

**Authors:** Kwang Ha

**Affiliations:** a School of Applied Chemical Engineering, The Research Institute of Catalysis, Chonnam National University, Gwangju 500-757, Republic of Korea

## Abstract

In the title complex, [PtI_2_(C_12_H_8_N_2_)], the Pt^2+^ ion is four-coordinated in a slightly distorted square-planar environment by two N atoms of the chelating 1,10-phenanthroline ligand and two iodide ions. The nearly planar mol­ecules, with a maximum deviation of 0.170 (3) Å from the least-squares plane, are stacked in columns along the *c* axis with a Pt⋯Pt distance of 4.8510 (6) Å. In the column, π–π inter­actions between adjacent six-membered rings are present, the shortest centroid–centroid distance being 3.703 (5) Å.

## Related literature

For the syntheses of [Pt*X*_2_(phen)] (phen = 1,10-phenanthroline; *X* = Cl, Br or I), see: Hodges & Rund (1975 ▶). For the crystal structure of yellow [PtCl_2_(phen)] which is isotypic to the title complex, see: Grzesiak & Matzger (2007 ▶).
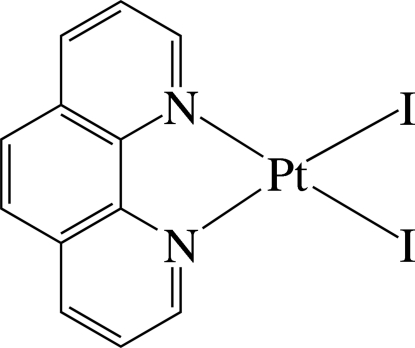


## Experimental

### 

#### Crystal data

[PtI_2_(C_12_H_8_N_2_)]
*M* *_r_* = 629.09
Monoclinic, 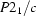

*a* = 10.3284 (9) Å
*b* = 17.9462 (16) Å
*c* = 7.3833 (7) Å
β = 108.569 (2)°
*V* = 1297.3 (2) Å^3^
*Z* = 4
Mo *K*α radiation
μ = 15.55 mm^−1^
*T* = 200 K
0.32 × 0.13 × 0.08 mm

#### Data collection

Bruker SMART 1000 CCD diffractometer
Absorption correction: multi-scan (*SADABS*; Bruker, 2000 ▶) *T*_min_ = 0.422, *T*_max_ = 1.000
7331 measured reflections
2284 independent reflections
2110 reflections with *I* > 2σ(*I*)
*R*_int_ = 0.033

#### Refinement

*R*[*F* ^2^ > 2σ(*F* ^2^)] = 0.033
*wR*(*F* ^2^) = 0.084
*S* = 1.06
2284 reflections
154 parameters
H-atom parameters constrained
Δρ_max_ = 3.05 e Å^−3^
Δρ_min_ = −1.40 e Å^−3^

### 

Data collection: *SMART* (Bruker, 2000 ▶); cell refinement: *SAINT* (Bruker, 2000 ▶); data reduction: *SAINT*; program(s) used to solve structure: *SHELXS97* (Sheldrick, 2008 ▶); program(s) used to refine structure: *SHELXL97* (Sheldrick, 2008 ▶); molecular graphics: *ORTEP-3* (Farrugia, 1997 ▶) and *PLATON* (Spek, 2009 ▶); software used to prepare material for publication: *SHELXL97*.

## Supplementary Material

Crystal structure: contains datablocks global, I. DOI: 10.1107/S1600536809053100/ng2700sup1.cif

Structure factors: contains datablocks I. DOI: 10.1107/S1600536809053100/ng2700Isup2.hkl

Additional supplementary materials:  crystallographic information; 3D view; checkCIF report

## Figures and Tables

**Table:** 

Pt1—N2	2.039 (6)
Pt1—N1	2.060 (7)
Pt1—I2	2.5774 (7)
Pt1—I1	2.5847 (6)

**Table:** 

N2—Pt1—N1	80.6 (2)

**Fig. 1.**
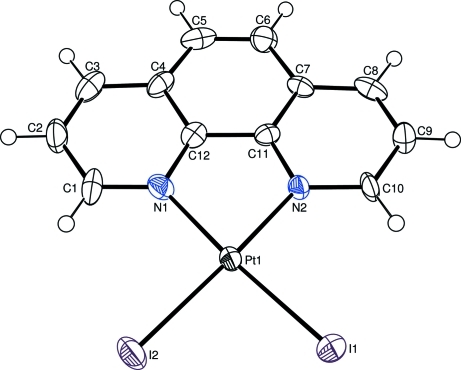
The structure of the title complex, with displacement ellipsoids drawn at the 50% probability level for non-H atoms.

**Fig. 2.**
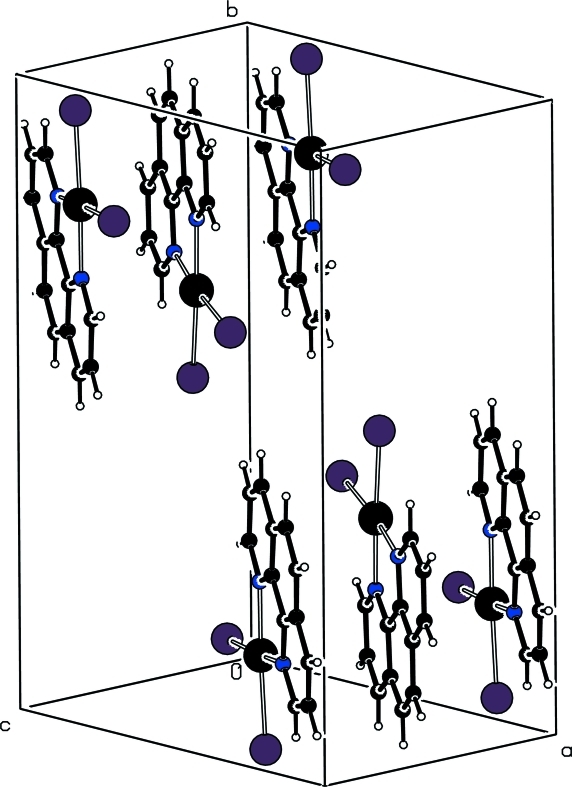
Crystal packing of the title complex.

## supplementary crystallographic information

### Comment

The title complex, [PtI_2_(phen)] (where phen is 1,10-phenanthroline, C_12_H_8_N_2_), is isomorphous with the yellow form of [PtCl_2_(phen)], whereas the orange form of [PtCl_2_(phen)] crystallized in the orthorhombic space group *Pca*2_1_ (Grzesiak & Matzger, 2007).

In the title complex, the Pt^2+^ ion is four-coordinated in a slightly distorted square-planar environment by two N atoms of the chelating 1,10-phenanthroline ligand and two iodide ions (Fig. 1). The main contribution to the distortion is the tight N1—Pt1—N2 chelate angle [80.6 (2)°], which results in non-linear *trans* arrangement [<N1—Pt1—I1 = 175.72 (17)° and <N2—Pt1—I2 = 175.02 (17)°]. The Pt1—N and Pt1—I bond lengths are almost equal, respectively [Pt1—N: 2.060 (7) and 2.039 (6) Å; Pt1—I 2.5847 (6) and 2.5774 (7) Å] (Table 1). The complex displays numerous intermolecular π-π interactions between adjacent six-membered rings, with a shortest centroid-centroid distance of 3.703 (5) Å and the dihedral angle between the ring planes is 3.4 (4)°. The nearly planar [PtI_2_(phen)] molecules, with a largest deviation of 0.170 (3) Å from the least-squares plane, stack in columns along the *c* axis with a Pt···Pt distance of 4.8510 (6) Å (Fig. 2).

### Experimental

To a solution of K_2_PtCl_4_ (0.2011 g, 0.484 mmol) in H_2_O (20 ml) were added KI (1.6010 g, 9.644 mmol) and 1,10-phenanthroline (0.0967 g, 0.537 mmol), and refluxed for 3 h. The precipitate obtained was separated by filtration, washed with water and acetone, and dried at 100 °C, to give a dark yellow powder (0.2732 g). Crystals suitable for X-ray analysis were obtained by slow evaporation from an *N,N*-dimethylformamide solution at 50 °C.

### Refinement

H atoms were positioned geometrically and allowed to ride on their respective parent atoms [C—H = 0.95 Å and *U*_iso_(H) = 1.2*U*_eq_(C)]. The maximum and minimum residual electron density peaks of 3.05 and -1.40 e Å^-3^, respectively, were located 0.97 and 0.92 Å from the Pt Atom.

### Crystal data

**Table:**

[PtI_2_(C_12_H_8_N_2_)]	*F*(000) = 1112
*M**_r_* = 629.09	*D*_x_ = 3.221 Mg m^−^^3^
Monoclinic, *P*2_1_/*c*	Mo *K*α radiation, λ = 0.71073 Å
Hall symbol: -P 2ybc	Cell parameters from 5463 reflections
*a* = 10.3284 (9) Å	θ = 2.3–28.2°
*b* = 17.9462 (16) Å	µ = 15.55 mm^−^^1^
*c* = 7.3833 (7) Å	*T* = 200 K
β = 108.569 (2)°	Needle, yellow
*V* = 1297.3 (2) Å^3^	0.32 × 0.13 × 0.08 mm
*Z* = 4	

### Data collection

**Table:**

Bruker SMART 1000 CCD diffractometer	2284 independent reflections
Radiation source: fine-focus sealed tube	2110 reflections with *I* > 2σ(*I*)
graphite	*R*_int_ = 0.033
φ and ω scans	θ_max_ = 25.1°, θ_min_ = 2.1°
Absorption correction: multi-scan (*SADABS*; Bruker, 2000)	*h* = −11→12
*T*_min_ = 0.422, *T*_max_ = 1.000	*k* = −21→21
7331 measured reflections	*l* = −8→8

### Refinement

**Table:**

Refinement on *F*^2^	Primary atom site location: structure-invariant direct methods
Least-squares matrix: full	Secondary atom site location: difference Fourier map
*R*[*F*^2^ > 2σ(*F*^2^)] = 0.033	Hydrogen site location: inferred from neighbouring sites
*wR*(*F*^2^) = 0.084	H-atom parameters constrained
*S* = 1.06	*w* = 1/[σ^2^(*F*_o_^2^) + (0.0524*P*)^2^ + 0.9657*P*] where *P* = (*F*_o_^2^ + 2*F*_c_^2^)/3
2284 reflections	(Δ/σ)_max_ < 0.001
154 parameters	Δρ_max_ = 3.05 e Å^−^^3^
0 restraints	Δρ_min_ = −1.40 e Å^−^^3^

### Special details

**Table:**

Geometry. All e.s.d.'s (except the e.s.d. in the dihedral angle between two l.s. planes) are estimated using the full covariance matrix. The cell e.s.d.'s are taken into account individually in the estimation of e.s.d.'s in distances, angles and torsion angles; correlations between e.s.d.'s in cell parameters are only used when they are defined by crystal symmetry. An approximate (isotropic) treatment of cell e.s.d.'s is used for estimating e.s.d.'s involving l.s. planes.
Refinement. Refinement of *F*^2^ against ALL reflections. The weighted *R*-factor *wR* and goodness of fit *S* are based on *F*^2^, conventional *R*-factors *R* are based on *F*, with *F* set to zero for negative *F*^2^. The threshold expression of *F*^2^ > σ(*F*^2^) is used only for calculating *R*-factors(gt) *etc*. and is not relevant to the choice of reflections for refinement. *R*-factors based on *F*^2^ are statistically about twice as large as those based on *F*, and *R*- factors based on ALL data will be even larger.

### Fractional atomic coordinates and isotropic or equivalent isotropic displacement parameters (Å^2^)

**Table:**

	*x*	*y*	*z*	*U*_iso_*/*U*_eq_	
Pt1	0.71220 (3)	0.337678 (16)	0.39502 (4)	0.02011 (13)	
I1	0.74244 (6)	0.48072 (3)	0.41617 (8)	0.03146 (17)	
I2	0.45788 (6)	0.35783 (3)	0.20604 (9)	0.03744 (18)	
N1	0.7037 (6)	0.2230 (4)	0.3858 (8)	0.0245 (15)	
N2	0.9082 (6)	0.3142 (4)	0.5563 (9)	0.0219 (13)	
C1	0.6012 (9)	0.1789 (5)	0.2965 (12)	0.034 (2)	
H1	0.5167	0.2005	0.2237	0.041*	
C2	0.6130 (9)	0.1009 (5)	0.3056 (13)	0.035 (2)	
H2	0.5376	0.0709	0.2367	0.042*	
C3	0.7305 (10)	0.0681 (6)	0.4114 (11)	0.036 (2)	
H3	0.7375	0.0153	0.4195	0.044*	
C4	0.8426 (9)	0.1132 (4)	0.5097 (12)	0.0291 (19)	
C5	0.9726 (10)	0.0861 (5)	0.6267 (13)	0.034 (2)	
H5	0.9862	0.0338	0.6419	0.041*	
C6	1.0755 (9)	0.1317 (5)	0.7155 (13)	0.0317 (19)	
H6	1.1602	0.1114	0.7921	0.038*	
C7	1.0598 (8)	0.2110 (4)	0.6964 (11)	0.0248 (17)	
C8	1.1647 (8)	0.2622 (5)	0.7825 (11)	0.0311 (19)	
H8	1.2519	0.2453	0.8600	0.037*	
C9	1.1388 (9)	0.3359 (4)	0.7527 (12)	0.0300 (19)	
H9	1.2091	0.3708	0.8101	0.036*	
C10	1.0137 (8)	0.3614 (5)	0.6417 (11)	0.0261 (17)	
H10	0.9999	0.4135	0.6236	0.031*	
C11	0.9351 (8)	0.2394 (4)	0.5838 (10)	0.0208 (16)	
C12	0.8236 (8)	0.1915 (4)	0.4917 (11)	0.0233 (17)	

### Atomic displacement parameters (Å^2^)

**Table:**

	*U*^11^	*U*^22^	*U*^33^	*U*^12^	*U*^13^	*U*^23^
Pt1	0.0168 (2)	0.0209 (2)	0.0209 (2)	−0.00133 (10)	0.00349 (14)	−0.00034 (10)
I1	0.0309 (3)	0.0217 (3)	0.0393 (3)	0.0010 (2)	0.0077 (3)	0.0001 (2)
I2	0.0184 (3)	0.0439 (4)	0.0433 (4)	0.0001 (2)	0.0004 (2)	0.0026 (3)
N1	0.024 (4)	0.028 (4)	0.023 (3)	−0.004 (3)	0.010 (3)	−0.002 (3)
N2	0.018 (4)	0.025 (3)	0.022 (3)	0.001 (3)	0.006 (3)	0.001 (3)
C1	0.031 (5)	0.040 (5)	0.032 (5)	−0.019 (4)	0.010 (4)	−0.012 (4)
C2	0.030 (5)	0.034 (5)	0.041 (5)	−0.015 (4)	0.011 (4)	−0.007 (4)
C3	0.059 (7)	0.026 (5)	0.031 (5)	−0.006 (4)	0.025 (5)	−0.005 (3)
C4	0.043 (5)	0.021 (4)	0.031 (4)	−0.005 (4)	0.023 (4)	0.001 (3)
C5	0.048 (6)	0.024 (5)	0.038 (5)	0.009 (4)	0.023 (5)	0.002 (4)
C6	0.028 (5)	0.032 (5)	0.038 (5)	0.003 (4)	0.014 (4)	0.004 (4)
C7	0.019 (4)	0.030 (4)	0.027 (4)	0.007 (3)	0.009 (3)	0.003 (3)
C8	0.020 (4)	0.046 (5)	0.025 (4)	0.010 (4)	0.005 (3)	0.006 (4)
C9	0.026 (5)	0.033 (5)	0.025 (4)	−0.005 (3)	0.000 (4)	−0.001 (3)
C10	0.017 (4)	0.023 (4)	0.032 (4)	−0.008 (3)	−0.001 (3)	−0.003 (3)
C11	0.024 (4)	0.019 (4)	0.021 (4)	−0.003 (3)	0.010 (3)	0.000 (3)
C12	0.033 (5)	0.021 (4)	0.022 (4)	−0.001 (3)	0.016 (4)	0.003 (3)

### Geometric parameters (Å, °)

**Table:**

Pt1—N2	2.039 (6)	C4—C12	1.419 (11)
Pt1—N1	2.060 (7)	C4—C5	1.431 (13)
Pt1—I2	2.5774 (7)	C5—C6	1.337 (12)
Pt1—I1	2.5847 (6)	C5—H5	0.9500
N1—C1	1.319 (10)	C6—C7	1.434 (11)
N1—C12	1.360 (10)	C6—H6	0.9500
N2—C10	1.365 (10)	C7—C11	1.389 (11)
N2—C11	1.374 (10)	C7—C8	1.407 (12)
C1—C2	1.405 (13)	C8—C9	1.354 (12)
C1—H1	0.9500	C8—H8	0.9500
C2—C3	1.351 (13)	C9—C10	1.369 (12)
C2—H2	0.9500	C9—H9	0.9500
C3—C4	1.410 (12)	C10—H10	0.9500
C3—H3	0.9500	C11—C12	1.423 (11)
			
N2—Pt1—N1	80.6 (2)	C6—C5—C4	122.3 (8)
N2—Pt1—I2	175.02 (17)	C6—C5—H5	118.9
N1—Pt1—I2	95.57 (17)	C4—C5—H5	118.9
N2—Pt1—I1	95.23 (18)	C5—C6—C7	120.8 (8)
N1—Pt1—I1	175.72 (17)	C5—C6—H6	119.6
I2—Pt1—I1	88.63 (2)	C7—C6—H6	119.6
C1—N1—C12	118.5 (8)	C11—C7—C8	117.7 (7)
C1—N1—Pt1	129.5 (6)	C11—C7—C6	118.5 (7)
C12—N1—Pt1	112.1 (5)	C8—C7—C6	123.8 (7)
C10—N2—C11	116.4 (7)	C9—C8—C7	118.7 (8)
C10—N2—Pt1	129.8 (5)	C9—C8—H8	120.6
C11—N2—Pt1	113.8 (5)	C7—C8—H8	120.6
N1—C1—C2	122.1 (9)	C8—C9—C10	121.5 (8)
N1—C1—H1	119.0	C8—C9—H9	119.2
C2—C1—H1	119.0	C10—C9—H9	119.2
C3—C2—C1	120.6 (8)	N2—C10—C9	122.1 (8)
C3—C2—H2	119.7	N2—C10—H10	118.9
C1—C2—H2	119.7	C9—C10—H10	118.9
C2—C3—C4	119.2 (9)	N2—C11—C7	123.5 (7)
C2—C3—H3	120.4	N2—C11—C12	115.2 (7)
C4—C3—H3	120.4	C7—C11—C12	121.3 (7)
C3—C4—C12	117.0 (8)	N1—C12—C4	122.6 (7)
C3—C4—C5	125.0 (8)	N1—C12—C11	118.2 (7)
C12—C4—C5	117.9 (7)	C4—C12—C11	119.1 (7)
			
N2—Pt1—N1—C1	−178.5 (7)	Pt1—N2—C10—C9	177.2 (6)
I2—Pt1—N1—C1	4.8 (7)	C8—C9—C10—N2	0.4 (13)
N2—Pt1—N1—C12	2.5 (5)	C10—N2—C11—C7	2.1 (10)
I2—Pt1—N1—C12	−174.3 (4)	Pt1—N2—C11—C7	−176.9 (6)
N1—Pt1—N2—C10	179.0 (7)	C10—N2—C11—C12	−179.5 (6)
I1—Pt1—N2—C10	0.1 (7)	Pt1—N2—C11—C12	1.6 (8)
N1—Pt1—N2—C11	−2.2 (5)	C8—C7—C11—N2	−1.6 (11)
I1—Pt1—N2—C11	178.8 (4)	C6—C7—C11—N2	179.6 (7)
C12—N1—C1—C2	−0.9 (11)	C8—C7—C11—C12	−179.9 (7)
Pt1—N1—C1—C2	−179.9 (6)	C6—C7—C11—C12	1.2 (10)
N1—C1—C2—C3	1.6 (12)	C1—N1—C12—C4	0.0 (10)
C1—C2—C3—C4	−1.4 (12)	Pt1—N1—C12—C4	179.2 (5)
C2—C3—C4—C12	0.5 (11)	C1—N1—C12—C11	178.4 (7)
C2—C3—C4—C5	179.9 (8)	Pt1—N1—C12—C11	−2.4 (8)
C3—C4—C5—C6	179.5 (7)	C3—C4—C12—N1	0.2 (10)
C12—C4—C5—C6	−1.1 (12)	C5—C4—C12—N1	−179.3 (7)
C4—C5—C6—C7	−0.2 (12)	C3—C4—C12—C11	−178.2 (7)
C5—C6—C7—C11	0.1 (11)	C5—C4—C12—C11	2.4 (10)
C5—C6—C7—C8	−178.6 (8)	N2—C11—C12—N1	0.6 (10)
C11—C7—C8—C9	0.4 (11)	C7—C11—C12—N1	179.1 (6)
C6—C7—C8—C9	179.2 (7)	N2—C11—C12—C4	179.0 (6)
C7—C8—C9—C10	0.1 (12)	C7—C11—C12—C4	−2.5 (10)
C11—N2—C10—C9	−1.5 (11)		
